# Medial or Lateral, That Is the Question: A Retrospective Study to Compare Two Injection Techniques in the Treatment of Knee Osteoarthritis Pain with Hyaluronic Acid

**DOI:** 10.3390/jcm13041141

**Published:** 2024-02-17

**Authors:** Giacomo Farì, Rachele Mancini, Laura Dell’Anna, Vincenzo Ricci, Simone Della Tommasa, Francesco Paolo Bianchi, Ilaria Ladisa, Carlo De Serio, Silvia Fiore, Danilo Donati, Maurizio Ranieri, Andrea Bernetti, Marisa Megna

**Affiliations:** 1 Department of Translational Biomedicine and Neuroscience, Aldo Moro University, 70121 Bari, Italymaurizio.ranieri@uniba.it (M.R.);; 2 Department of Biological and Environmental Science and Technologies, University of Salento, 73100 Lecce, Italy; 3 Physical and Rehabilitation Medicine Unit, Luigi Sacco University Hospital, 20121 Milano, Italy; 4 Department for Horses, University of Leipzig, 04103 Leipzig, Germany; 5 Interdisciplinary Department of Medicine, University of Bari, 70124 Bari, Italy; 6 School of Specialization in Rheumatology, Fondazione Polclinico Universitario Agostino Gemelli IRCCS, 00168 Roma, Italy; 7 Clinical and Experimental Medicine PhD Program, University of Modena and Reggio Emilia, 41121 Modena, Italy

**Keywords:** knee osteoarthritis, hyaluronic acid, intra-articular hyaluronic acid, medial infrapatellar approach, lateral infrapatellar approach, rehabilitation

## Abstract

**Background**: Mild-to-moderate knee osteoarthritis (KOA) can be successfully treated using intra-articular hyaluronic acid (IA-HA). The medial infrapatellar (MIP) approach and lateral infrapatellar (LIP) approach are two of the most used techniques for performing IA-HA, but it is still not clear which one is preferable. **Objectives**: The study aims to find the best knee injection technique between MIP and LIP approaches. Methods: In total, 161 patients were enrolled, divided into two groups (MIP or LIP). Each technique was performed once a week for three weeks. Patients were evaluated using the Numeric Rating Scale (NRS), Knee Injury and Osteoarthritis Outcome Score (KOOS) and Roles and Maudsley Score (RMS) at T0 (before the first injection), T1 (one week after the third injection) and T2 (six months after). **Results**: NRS, KOOS and RMS showed a statistically significant improvement in both groups at all the detection times, without significant differences. No differences were detected between the groups in terms of systemic effect effusions, while the MIP group presented a mildly higher number of bruises in comparison with the LIP group (*p* = 0.034). **Conclusions**: Both the IA-HA techniques are equally effective in measured outcomes. The MIP approach seems to produce some local and transient side effects. So, the choice of the LIP or MIP approach depends on the operator’s skill and experience.

## 1. Introduction

Osteoarthritis (OA) is a chronic degenerative articular disease [1,2,3]. Knee osteoarthritis (KOA) is the most frequent since the knee joint is particularly exposed to mechanical overloads [4], causing chronic pain and severe motor impairments, which lead to disability and loss of independence in carrying out the activities of daily living [5].

There are many therapies for KOA treatment, from drugs and new nutraceutical products to relieve pain [6,7] to prosthetic surgery during the most severe stages [8]. 

Hyaluronic acid (HA) is a glycosaminoglycan that occurs naturally in the knee synovial fluid. In KOA, due to decreased HA production, degradation and increased clearance, synovial fluid HA concentration is lower than in healthy knees. The aim of HA intra-articular injections (IA-HA) is the restoration of its viscoelastic properties, preventing cartilage degradation, promoting its regeneration and reducing chronic pain [9]. IA-HA represents a valid and effective option for mild-to-moderate KOA management, as well as for severe non-surgical management [10]. 

There are many approaches to performing knee IA-HA injections. Maricar et al. identified eight different knee injection sites for the palpation-guided technique. Nevertheless, physician experience largely influences the accuracy of injections. A high parapatellar approach is preferred for fluid evacuation, while two of the most used are the lateral infrapatellar (LIP) approach and the medial infrapatellar (MIP) one [11]. In both cases, the IA-HA injection is performed with the knee flexed at 90°, accessing the knee joint by passing next to the patella and the related tendon with the medial or lateral patellofemoral approach.

KOA most often affects the medial tibiofemoral compartment. As a consequence, patients frequently report pain located in the medial compartment of the knee [12]. Consequently, among patients, it is commonly thought that the MIP approach could be more effective due to the needle placement nearer the pain site.

Although previous studies have been conducted to evaluate the most effective needle placement into the knee intra-articular space, to our knowledge, none of the previous investigations compared these two approaches in terms of effectiveness and local side effects. 

Since there is still no evidence available that the medial approach grants better outcomes or whether one of the two techniques is more valid than the other, a retrospective study was carried out to compare the effectiveness of these two techniques in terms of clinical outcomes and local side effects for treating KOA with HA injections.

## 2. Materials and Methods

This study is an observational retrospective one. It was carried out according to the Declaration of Helsinki Principles, and it received the approval of the human ethical committee of the General Hospital of Bari, Italy, protocol number 1402/CEL, 13 December 2023.

The written informed consent of each enrolled subject was originally collected as an express acceptance to undergo the injection treatments and to allow the use of the data for scientific research purposes.

### 2.1. Study Population

We retrospectively enrolled 161 patients (74 men and 87 women) affected by KOA who attended the Physical Medicine and Rehabilitation outpatient service of the Bari General Hospital from January 2019 to March 2023.

The inclusion criteria were as follows:

- Diagnosis of KOA confirmed by a clinical medical evaluation and by an X-ray taken within the previous 12 months;
- Kellgren–Lawrence (KL) REF grade 2–3;
- Age between 55 and 75 years;
- Monolateral Knee Pain (NRS > 3) lasting for at least 3 months or longer.

The exclusion criteria were as follows:

- Previous knee surgery;
- Diagnosis of other musculoskeletal or neurological or rheumatological disorders affecting the lower limbs;
- Any KOA local and systemic treatment in the previous 6 months (therapeutic exercises, physical therapy, other injections, NSAIDs, etc.);
- Pharmacological therapies or systemic diseases which contraindicate injection treatments (e.g., anticoagulant drugs, coagulopathies).

The patients were retrospectively divided into two different groups according to the injection site. Group A consisted of 79 KOA patients treated once a week for three consecutive weeks with three HA MIP injections; group B consisted of 82 KOA patients treated with three HA LIP injections once a week for three consecutive weeks. All injections were performed by an expert physiatrist who had 5 years of experience in knee IA injections.

### 2.2. Intervention

**Figure 1:**
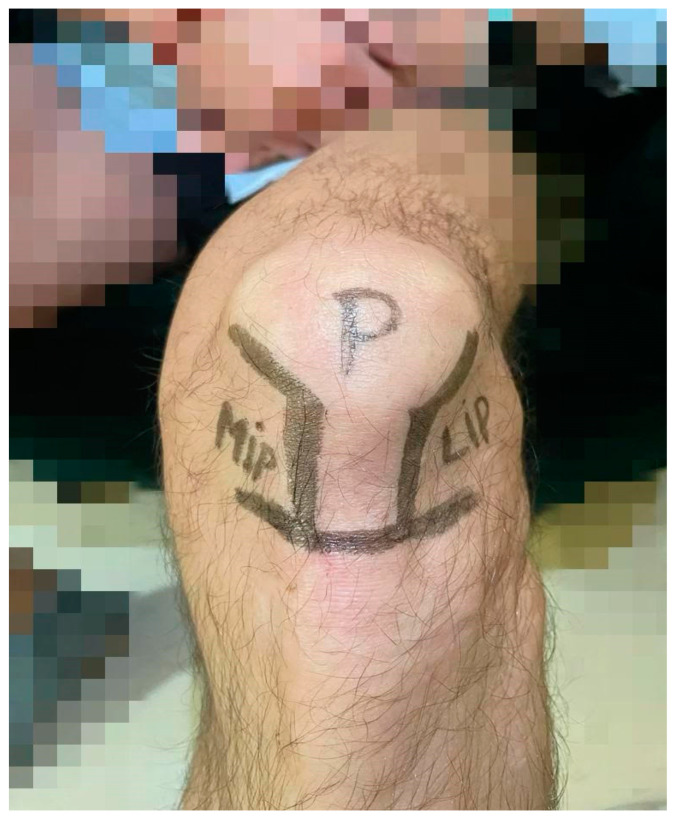
MIP and LIP knee joint injection access (left knee).

The patient was positioned supine with the hip flexed at approximately 45° and the knee flexed at approximately 45°. Before each injection, a meticulous skin disinfection was performed using sterile gauzes soaked in povidone iodine solution. The same high-molecular-weight (>1500 kDalton) HA was used for each injection using a 2.0 in (5.1 cm) 21-gauge needle. Each vial contained 30 mg of HA in 2 mL. The performed injection techniques were the standard LIP and MIP techniques delivered in an ultrasound-assisted way (Figure 1).

**Figure 2:**
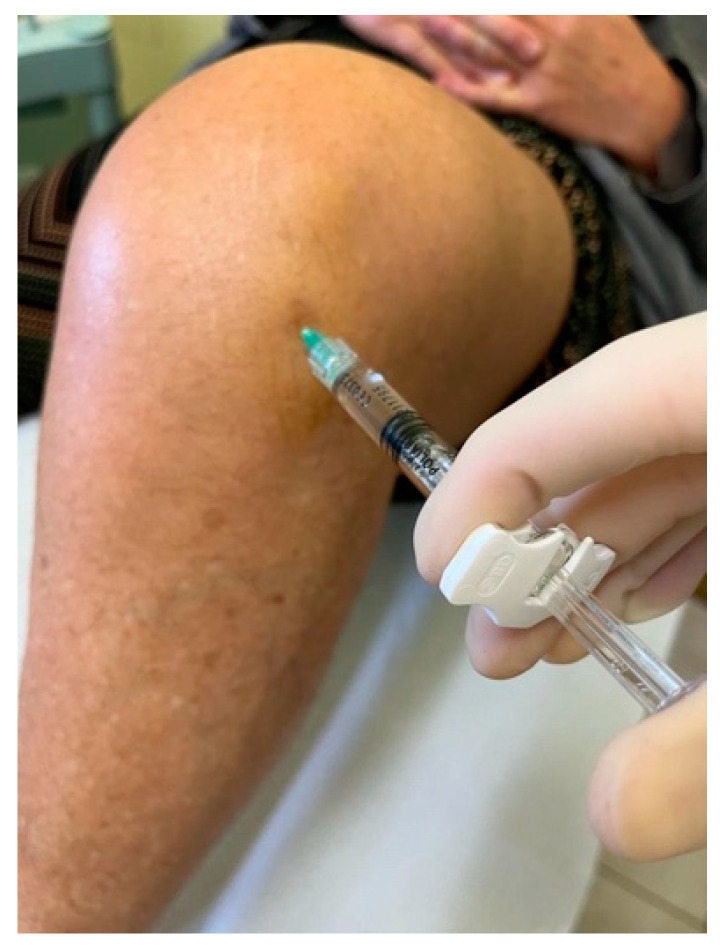
LIP technique performed in the left knee.

The preliminary ultrasound assessment is useful for evaluating the anatomical structures and for establishing the correct needle direction [13]. In the LIP technique, the needle is inserted about 1 cm below and 1 cm lateral to the inferior lateral margin of the patella, and then it is directed diagonally, going from the lateral side behind the patella (Figure 2). 

**Figure 3:**
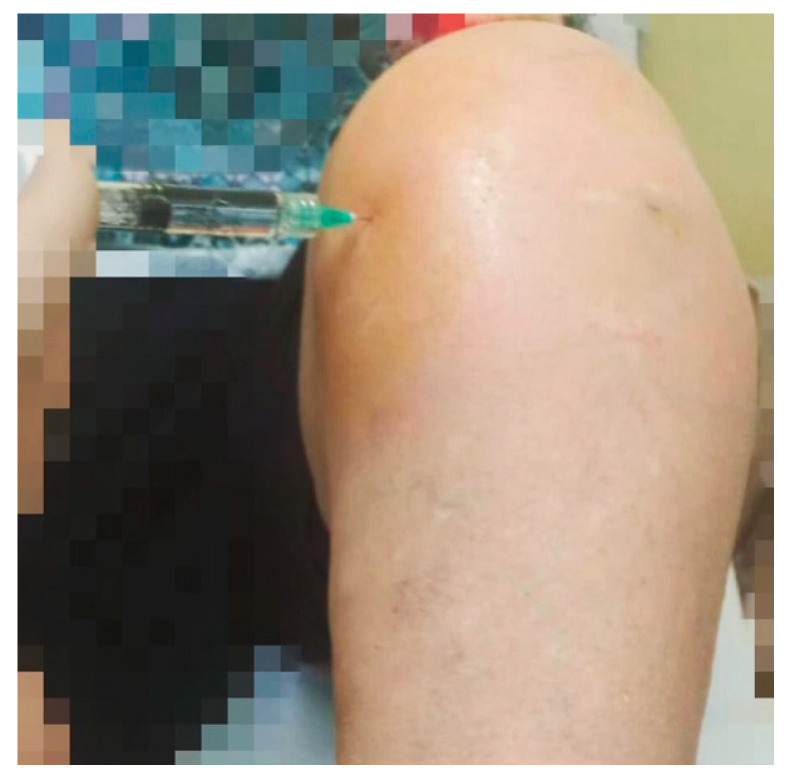
MIP technique performed in the left knee.

In the MIP technique, the needle is inserted about 1 cm below and 1 cm medially to the inferior medial aspect of the patella, and then it is directed obliquely, going from the medial side behind the patella (Figure 3).

### 2.3. Timing

All the involved patients were evaluated by three different physiatrists at the following detection times:

- T0: at the enrolment, which overlapped with the date of the first injection;
- T1: one week after completing the IA-HA cycle, three weeks after the first injection;
- T2: six months after the first IA-HA injection.

The first injection was administered at T0, the second one a week after the first and the third one a week after the second.

### 2.4. Outcome Measures

The aim of this study was to assess the best clinical knee injection approach between MIP and LIP techniques in terms of knee pain and functional improvement and local side effects. At T0, T1 and T2, after a medical and ultrasound evaluation of the treated knee, each patient was evaluated with the Numeric Rating Scale (NRS) and Knee Injury and Osteoarthritis Outcome Score (KOOS). The NRS is a validated pain scale with a score ranging from 0 (no pain) to 10 (maximum pain) [14]. The KOOS is a 42-item questionnaire useful for assessing self-reported progress in knee functions [15]. At T1 and T2, the Roles and Maudsley Score (RMS) was also collected for each patient. The RMS is a subjective patient assessment of pain, and it was used as an instrument to evaluate the satisfaction with the treatment in terms of effectiveness, discomfort related to the execution and the procedure’s side effects. At T1 and T2, for each patient, the examining physiatrist filled out a diary with all the local effects reported after the injections. The side effects were recorded after an inspection and palpatory examination to highlight bruises, hematomas and sites of pain; then, they were further investigated with an ultrasound evaluation.

### 2.5. Statistical Analysis

Data were analyzed using Stata MP18 software. We expressed continuous variables as mean ± standard deviation (SD) and range; categorical data were expressed as proportions. The Skewness and Kurtosis test was used to compare normal distribution of continuous data, and, whenever possible, we created a normal model for those data not normally distributed. For parametric data, a Student’s *t*-test was used to compare continuous variables between the two groups. The continuous variables were compared between the two groups using the Student’s *t*-test for independent data or using the Wilcoxon signed-rank test for non-parametric data. An ANOVA test was used for repetitive measures, comparing different timing. The Chi-squared test was used to compare categorical variables between groups. A multivariate linear regression was used for the analysis of the relationship between the NRS and KOOS outcomes at T0 and T2, and between sex, age, BMI and groups. Confidence interval was set at 95%, while a *p*-value was considered statistically significant if <0.05.

## 3. Results

The study sample was made up of 161 (74 men and 87 women) patients suffering from KOA, divided into group A, composed of 79 people (49.1%), and group B, composed of 82 subjects (59.9%). The average age was 66 ± 4.3 years (range 56–75). The groups were homogenous regarding age, gender and BMI. The sample’s characteristics are resumed in Table 1.

The outcome variables, by group and detection time, are described in Table 2. The NRS showed a statistically significant reduction in subsequent time points in both groups (group A: T1 6.6 ± 1.0 (4–8), T2 2.5 ± 0.8 (1–5), T3 2.2 ± 0.7 (0–4); group B: T1 6.7 ± 1.0 (4–8), T2 2.2 ± 0.7 (0–4), T3 2.0 ± 0.7 (0–4)). In all the KOOS scale’s sections, values increased between T0 and T1 and T2 in group A as well as in group B in a statistically significant way (*p* < 0.0001). All these findings are fully described in Table 2. 

**Figure 4:**
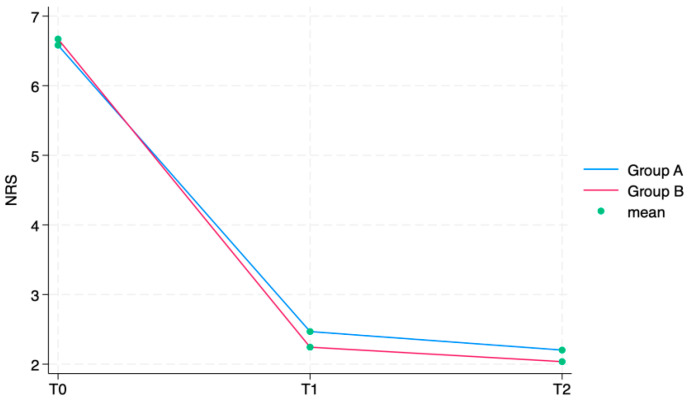
NRS by group and detection time.

**Figure 5:**
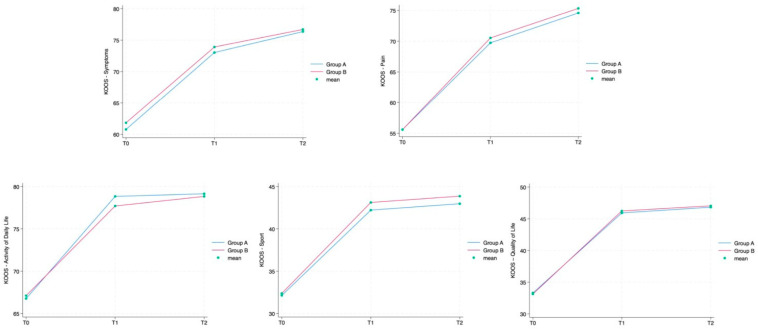
KOOS scale—Symptoms, Pain, Activity of Daily Life, Sport, Quality of Life—by group.

**Figure 6:**
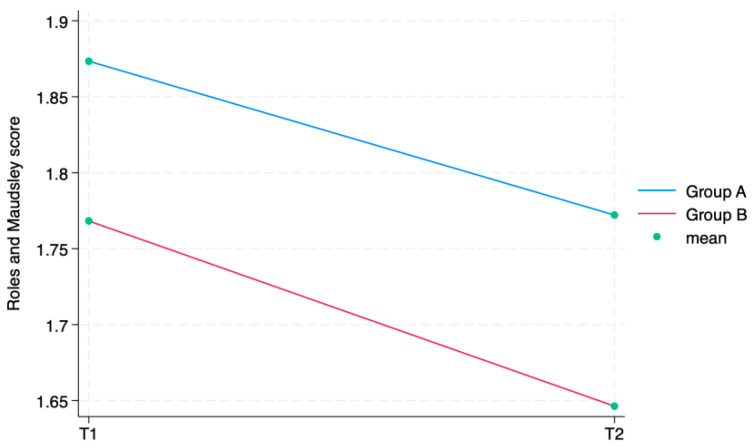
Roles and Maudsley Score, by group.

The ANOVA test for repeated measurements showed a statistically significant difference for all the aforementioned outcome measures in the comparison between times. The Roles and Maudsley Score, assessing procedure satisfaction as a self-reported outcome, showed a minimum decrement between T1 and T2 and a difference between groups (*p* = 0.042), both not statistically relevant. Every single outcome is also visually represented as line graphs in Figure 4 and Figure 5, showing the evolution over time for each scale. Figure 6 displays the Roles and Maudsley Score at each evaluation time. The figure underlines the difference between the two groups, but, as outlined in Table 2, the *p*-value is >0.05; therefore, it is not statistically significant.

Table 3, Table 4, Table 5, Table 6, Table 7 and Table 8 describe the multivariate linear regression analyses by single outcome. Sex, age, BMI and knee side were identified as potential confounders and included in the multivariate linear regression analysis to investigate any influence on every single outcome measure.

In Table 9, the side effect prevalence is explained. No systemic adverse events and no allergic reactions (skin rash, hives) were reported. Only local adverse events were recorded, such as bruises and effusions. The presence of bruises was observed in 23 subjects (14.3%), and there was a statistically significant difference between groups (group A: 16, 20.3% vs. group B: 7, 8.5%; *p*-value = 0.034). For 23 (14.3%) patients, there was effusion, without statistically significant differences between the groups (group A: 15, 19.0% vs. group B: 8, 9.8%; *p*-value = 0.094).

## 4. Discussion

The efficacy of IA-HA injections for treating KOA is already well known [16]. In fact, it represents a simple and safe procedure which grants short- and medium-term pain relief with a positive effect on joint functionality. Currently, there is weak evidence in the literature about the long-term effects of IA-HA for pain relief, but some studies demonstrated that IA-HA can delay knee arthroplasty surgery [17].

The best approach for knee injection is still uncertain; the procedure choice is often based only on the physician’s experience. The goal is to deliver an adequate quote of medication in the IA space, to improve the technique accuracy and to reduce the risk that, during the injection, the needle may engage with the medial knee plica or the fat pad.

Our findings demonstrated the efficacy of IA-HA. In fact, both groups significantly improved between T0 and T2 both in terms of pain reduction according to NRS scores (*p* < 0.0001) and in terms of joint function, according to KOOS (*p* < 0.0001). 

Particularly, the NRS decreased by approximately four points in both groups. These results are in line with the current scientific literature, which states the effectiveness of IA-HA injections in relieving knee pain during up to 6 months of follow up [18]. Similarly, KOOS values improved in both groups and for each scale section. Also, these results are in line with the available evidence of IA-HA injections’ effectiveness [19,20]. 

The multivariate linear regression analysis ruled out that these results were influenced by the determinants described in Table 1, except for two aspects. Particularly, sex seemed to slightly affect the NRS scores (*p* = 0.024) so that females seemed to have a better response to IA-HA. This gap may be due to a different experience of pain between men and women. NRS values slightly differ among people. Furthermore, women experience pain more frequently due to the role of sex and gender; this aspect could refer to many causes, ranging from factors related to biological sex to those related to psychosocial gender. Moreover, to our knowledge, the previous literature never reported or investigated gender differences in IA-HA effects, and it would be desirable for this aspect to be explored in future studies. The other apparently significant determinant is the BMI with respect to the trend of the KOOS Symptoms score (*p* = 0.025). In this case, an explanation could be the fact that people with a higher BMI usually have a lower KOOS Symptoms rate at baseline, and, therefore, they obtain a more marked increase in KOOS Symptoms rate between T0 and T2 due to the benefits derived from IA-HA. 

No weight-related difference was found in pain scale between the two groups. Partially in contrast with our results, a study conducted by D’Alessandro et al. compared the accuracy of other injection techniques in overweight patients (BMI > 25) affected by KOA. They found no variation of injection-related pain in IA-HA between anterolateral and superolateral access. According to their research, an increase in BMI seems to be indicative of greater pain during anterolateral access. They explained this evidence as a consequence of a greater local production of adipocytokines, due to the augmented subcutaneous tissue in overweight patients, rather than to Hoffa’s fat pad, whose volume seems to not be related to weight [21]. 

Most importantly for our research, there were no significant differences between the two groups according to NRS and KOOS. Based on our results, MIP and LIP approaches are equally effective as minimally invasive KOA treatments so neither approach is preferable to the other. 

In the available scientific literature, data are lacking in the specific comparison between MIP and LIP approaches. A comparison study by Toda et al. [22] deepened the accuracy rates of three different knee IA approaches, namely, LIP and MIP approaches with the patient in a seated position and the modified Waddell approach, an anteromedial approach with manipulative ankle traction at 30 degrees of knee flexion. Although the number of patients was small, no significant differences were detected between the three techniques for KL 2 and 3 patients (*p* > 0.05), in line with our results.

The anterolateral approach, both medial and lateral, seems to be more accurate and effective than the traditional superolateral one [23]. In fact, the MIP and LIP techniques are useful when the knee is dry, without joint effusions, with no anatomical variations, and when the knee cannot be fully extended [24]. Moreover, these approaches are easy to perform also with a palpatory landmark guide in the absence of an ultrasound guide [25].

Regarding RMS, at each detection time, both groups had a high satisfaction with the received injection treatment. Even though there was no statistically significant difference between the groups, we observed an interesting trend in favor of the LIP approach (*p* = 0.051). Although this is only a trend, this can probably be justified by the fact that the LIP technique seems to be more accurate when the knee is dry; therefore, with the same effectiveness, it can be less painful for patients as it allows for a more accurate infiltration [26]. In fact, a study by Jackson et al. compared different IA knee injections using real-time fluoroscopic imaging with contrast material and affirmed that a lateral midpatellar injection (an injection into the patellofemoral joint) was the most precise one since it was intra-articular in 93% of cases [27]. This finding is also validated in a paper by Park et al. that investigated the injection accuracy rate in three different knee sites with an ultrasound-guided approach. They stated that, in KOA, ultrasound-guided IA-HA injections in the mediolateral or superolateral space were more accurate than those through the medial space [28].

This reasoning could be extended also to the analysis of the findings regarding the local side effects. In fact, there were no systemic adverse effects, while the results obtained for bruises and joint effusions were different in statistical terms. No differences were detected between the groups in terms of effusions, while the MIP group (group A) presented a significantly higher number of bruises in comparison with the LIP group (*p* = 0.034). The higher frequency of bruises could be due to the fact that, in the MIP approach, there is a higher risk of crossing subcutaneous small veins. Our results are in line with a previous study published by Lussier et al. [29] that confirms that both techniques are safe, but the LIP one seems to be more accurate, making even minimal injection-related discomforts less frequent.

In conclusion, MIP and LIP techniques appear to be totally equivalent in terms of effectiveness. This evidence is far from obvious or without usefulness. On the contrary, it allows us to choose the approach based on the skills of the operator performing the injections or based on the clinical contingency. Sometimes, KOA determines an alteration of the joint anatomy and a deformity such as to force access from one side rather than another [30,31]. Particularly, the medial knee compartment is more frequently altered by KOA, and the bone reshaping could be an obstacle to a correct and easy injection using the MIP technique [32,33]. In these cases, the LIP approach could be preferred, as well as in cases where there is an increased risk of bleeding and bruising caused by the infiltration itself (for example, in patients taking anticoagulants). Similarly, when there are no preferences due to the physician’s expertise or due to specific anatomical contingencies, the LIP technique could be more advantageous due to a lower risk of even minimal side effects.

The current study presents some limitations. First of all, pain is a difficult parameter to assess in an objective way. In fact, pain outcomes were self-reported, but it was a mandatory condition to evaluate it. Then, in the literature, various knee entry sites are described for injecting HA, but, as we said above, we chose the two most used techniques. Therefore, further studies are needed to investigate the best injective way, including also other techniques and different operators. We established a relatively long-term follow up (6 months); however, it would be interesting to better understand long-term efficacy to investigate the differences in terms of injection frequency between the two techniques in a perspective study.

## 5. Conclusions

MIP and LIP techniques seem to be equally effective and safe as IA-HA injection procedures for patients suffering from chronic pain related to KOA. Therefore, the choice of the technique to be performed can be based on the operators’ practical experience, thus reducing the risk of side effects.

Anatomical variations and specific risk factors, such as coagulopathies, may make the execution of the LIP technique more suitable, just as the degree of patient satisfaction may require switching to one approach rather than another during the same injection cycle.

Further studies are needed to deepen these aspects and to continuously refine knee infiltration techniques in order to increase the patients’ satisfaction and compliance with therapies.

## Figures and Tables

**Table 1:** Characteristics of the sample divided per group.

Parameter	Group A	Group B	Total	*p*-Value
Age (years); mean ± SD (range)	65.8 ± 4.3 (56–73)	66.3 ± 4.4 (57–75)	66.0 ± 4.3 (56–75)	0.481
Male; *n* (%)	35 (44.3)	39 (47.6)	74 (46.0)	0.678
BMI (kg/m^2^); mean ± SD (range)	27.5 ± 2.6 (22–35)	27.2 ± 2.5 (23–36)	27.4 ± 2.5 (22–36)	0.249
Side; *n* (%)				0.402
left	35 (44.3)	31 (37.8)	66 (41.0)	
right	44 (55.7)	51 (62.2)	95 (59.0)	
Doppler activity; *n* (%)	6 (7.6)	9 (11.0)	15 (9.3)	0.461

BMI = body mass index; SD = standard deviation.

**Table 2:** Average ± SD and range of outcomes per time and group.

	T0	T1	T2	Group Comparison	Time Comparison	Time and Group Interaction
NRS						
Group A	6.6 ± 1.0 (4–8)	2.5 ± 0.8 (1–5)	2.2 ± 0.7 (0–4)	0.283	<0.0001	0.117
Group B	6.7 ± 1.0 (4–8)	2.2 ± 0.7 (0–4)	2.0 ± 0.7 (0–4)			
Total	6.6 ± 1.0 (4–8)	2.4 ± 0.8 (0–5)	2.1 ± 0.7 (0–4)			
KOOS—Symptoms						
Group A	60.8 ± 5.6	73.0 ± 6.9	76.4 ± 5.8	0.348	<0.0001	0.770
	(50.1–71.0)	(54.6–89.0)	(64.7–91.0)			
Group B	61.8 ± 5.0	73.9 ± 7.5	76.7 ± 6.9			
	(52.4–72.0)	(57.0–88.0)	(60.4–90.0)			
Total	61.3 ± 5.3	73.5 ± 7.2	76.5 ± 6.4			
	(50.1–72.0)	(54.6–89.0)	(60.4–91.0)			
KOOS—Pain						
Group A	55.6 ± 6.2 (40–72)	69.7 ± 7.5 (53–88)	74.6 ± 7.8 (60–94)	0.528	<0.0001	0.756
Group B	55.6 ± 7.3 (39–73)	70.5 ± 6.5 (54–84)	75.3 ± 5.0 (60–92)			
Total	55.6 ± 6.8 (39–73)	70.1 ± 7.0 (53–88)	75.0 ± 6.5 (60–94)			
KOOS—Activity of Daily Life						
Group A	66.8 ± 7.4	78.8 ± 6.3	79.1 ± 6.7	0.628	<0.0001	0.398
	(46.5–83.4)	(61.0–95.0)	(65.0–90.2)			
Group B	67.1 ± 5.4	77.7 ± 6.2	78.8 ± 5.4			
	(48.8–87.3)	(63.6–95.0)	(64.0–90.0)			
Total	66.9 ± 6.4	78.3 ± 6.2	79.0 ± 6.1			
	(46.5–87.3)	(61.0–95.0)	(64.0–90.2)			
KOOS—Sport						
Group A	32.1 ± 6.0	42.2 ± 5.7	43.0 ± 5.4	0.354	<0.0001	0.633
	(16–50)	(22.0–52.0)	(28–51)			
Group B	32.4 ± 5.0	43.1 ± 5.4	43.9 ± 5.4			
	(17–48)	(26–51)	(28–54)			
Total	32.3 ± 5.5	42.7 ± 5.6	43.4 ± 5.4			
	(16–50)	(22–52)	(28–54)			
KOOS—Quality of Life						
Group A	33.3 ± 5.0	45.9 ± 5.7	46.8 ± 5.0	0.852	<0.0001	0.854
	(18–48)	(25–65)	(25–66)			
Group B	33.1 ± 4.9	46.2 ± 5.8	47.0 ± 4.6			
	(17–48)	(25–66)	(40–65)			
Total	33.2 ± 4.9	46.1 ± 5.7	46.9 ± 4.8			
	(17–48)	(25–66)	(25–66)			
Roles and Maudsley Score						
Group A	-	1.9 ± 0.5	1.8 ± 0.5	0.051	0.042	0.849
		(1–3)	(1–3)			
Group B	-	1.8 ± 0.5	1.6 ± 0.5			
		(1–3)	(1–3)			
Total	-	1.8 ± 0.5	1.7 ± 0.5			
		(1–3)	(1–3)			

**Table 3:** A multivariate linear regression model to analyze the NRS variations between T2 and T0.

Variable	Coef.	95%CI	*p*-Value
Group (B vs. A)	−0.20	−0.52–0.13	0.238
Age (years)	−0.02	−0.06–0.16	0.248
Sex (male vs. female)	−0.38	−0.71–−0.05	0.024
BMI (kg/m^2^)	0.01	−0.05–0.08	0.658
Side (right vs. left)	−0.42	−0.76–0.09	0.014

BMI = body mass index; Coef. = coefficient; CI = confidence interval.

**Table 4:** A multivariate linear regression model to analyze the KOOS variations in Symptoms between T2 and T0.

Variable	Coef.	95%CI	*p*-Value
Group (B vs. A)	−0.72	−2.93–1.49	0.520
Age (years)	0.18	−0.07–0.44	0.157
Sex (male vs. female)	1.42	−0.79–3.64	0.206
BMI (kg/m^2^)	0.51	0.06–0.96	0.025
Side (right vs. left)	1.00	−1.26–3.26	0.386

BMI = body mass index; Coef. = coefficient; CI = confidence interval.

**Table 5:** A multivariate linear regression model to analyze the KOOS variations in Pain between T2 and T0.

Variable	Coef.	95%CI	*p*-Value
Group (B vs. A)	0.74	−1.89–3.38	0.578
Age (years)	0.24	−0.07–0.54	0.123
Sex (male vs. female)	0.41	−2.22–3.05	0.758
BMI (kg/m^2^)	0.25	−0.28–0.78	0.362
Side (right vs. left)	−0.34	−3.02–2.35	0.805

BMI = body mass index; Coef. = coefficient; CI = confidence interval.

**Table 6:** A multivariate linear regression model to analyze the KOOS variations in Activity of Daily Life between T2 and T0.

Variable	Coef.	95%CI	*p*-Value
Group (B vs. A)	−0.78	−3.27–1.71	0.536
Age (years)	−0.13	−0.42–0.15	0.363
Sex (male vs. female)	0.41	−2.08–2.90	0.747
BMI (kg/m^2^)	−0.30	−0.80–0.20	0.239
Side (right vs. left)	1.07	−1.47–3.60	0.408

BMI = body mass index; Coef. = coefficient; CI = confidence interval.

**Table 7:** A multivariate linear regression model to analyze KOOS variations in Sport between T2 and T0.

Variable	Coef.	95%CI	*p*-Value
Group (B vs. A)	0.69	−1.20–2.58	0.474
Age (years)	−0.01	−0.23–0.21	0.933
Sex (male vs. female)	0.99	−0.91–2.88	0.304
BMI (kg/m^2^)	0.22	−0.17–0.60	0.266
Side (right vs. left)	0.28	−1.64–2.21	0.774

BMI = body mass index; Coef. = coefficient; CI = confidence interval.

**Table 8:** A multivariate linear regression model to analyze KOOS variations in Quality of Life between T2 and T0.

Variable	Coef.	95%CI	*p*-Value
Group (B vs. A)	0.29	−1.73–2.31	0.778
Age (years)	0.13	−0.11–0.36	0.287
Sex (male vs. female)	0.38	−1.64–2.40	0.711
BMI (kg/m^2^)	−0.10	−0.50–0.31	0.641
Side (right vs. left)	−0.22	−2.28–1.83	0.834

BMI = body mass index; Coef. = coefficient; CI = confidence interval.

**Table 9:** Side effect prevalence (“bruise” and “effusion”) per group.

Variable	Group A (*n* = 79)	Group B (*n* = 82)	Total (*n* = 161)	*p*-Value
Effusion; *n* (%)	15 (19.0)	8 (9.8)	23 (14.3)	0.094
Bruise; *n* (%)	16 (20.3)	7 (8.5)	23 (14.3)	0.034

## Data Availability

The datasets used and/or analyzed during the current study will be made available upon reasonable request to the corresponding author (G.F.).
